# An Analog-Digital Mixed Measurement Method of Inductive Proximity Sensor

**DOI:** 10.3390/s16010030

**Published:** 2015-12-30

**Authors:** Yi-Xin Guo, Zhi-Biao Shao, Ting Li

**Affiliations:** The School of Electronic and Information Engineering, Xi’an Jiaotong University, No.28, Xianning West Road, Xi’an 710049, China; zbshao@mail.xjtu.edu.cn (Z.-B.S.); agike@sina.com (T.L.)

**Keywords:** inductive proximity sensor, analog-digital mixed measurement method, two-dimensional look-up table, built-in self-test, position detection

## Abstract

Inductive proximity sensors (IPSs) are widely used in position detection given their unique advantages. To address the problem of temperature drift, this paper presents an analog-digital mixed measurement method based on the two-dimensional look-up table. The inductance and resistance components can be separated by processing the measurement data, thus reducing temperature drift and generating quantitative outputs. This study establishes and implements a two-dimensional look-up table that reduces the online computational complexity through structural modeling and by conducting an IPS operating principle analysis. This table is effectively compressed by considering the distribution characteristics of the sample data, thus simplifying the processing circuit. Moreover, power consumption is reduced. A real-time, built-in self-test (BIST) function is also designed and achieved by analyzing abnormal sample data. Experiment results show that the proposed method obtains the advantages of both analog and digital measurements, which are stable, reliable, and taken in real time, without the use of floating-point arithmetic and process-control-based components. The quantitative output of displacement measurement accelerates and stabilizes the system control and detection process. The method is particularly suitable for meeting the high-performance requirements of the aviation and aerospace fields.

## 1. Introduction

Most proximity sensors use non-contact methods to detect the distance or the proximity event, and their advantages include a resistance to fouling and abrasion, water tightness, a long service life, and low mechanical system maintenance cost [1]; as well as a high mean time between failure (MTBF) value [2]; and strong magnetic immunity [3,4]. Comparing with capacitive proximity sensors, inductive proximity sensors (IPSs) have better sensitivity with a target of alloy steels. IPS is applicable in the industry field of position detection, especially in the aviation and aerospace fields [5].

The primary transducer of IPS is a coil, and its inductance component correlates to the distance between IPS and the target [1]. However, the resistance component of coil changes significantly as the temperature changes due to the effects of the coil structure and materials [1,6,7]. The resistance component also constrains the detection precision of IPS severely [8,9]. Besides, the traditional IPS detects the nearness of the target, but it cannot create quantitative output. Mizuno *et al.* [10] proposed the use of magneto-plated wire to fabricate coil because this wire can decrease AC resistance and reduce temperature drift [11,12]. However, this method cannot solve the temperature drift of the DC resistance element fundamentally. The performance of IPS depends on the coil structure and the processing circuit. In certain conditions, the greater the inductance component is, the better the IPS sensitivity will be. However, the temperature drift will be severe as the resistance component increases simultaneously, and modeling calculation will be complicated by the simultaneous increase in distributed capacitance [13]. The sensor signal processing circuit is important to the IPS performance [14]. Many methods have been developed to reduce temperature drift by improving the processing circuit, including the analog [6,15] and digital measurement methods [16].

Analog measurement procedures include applying pulse excitation to a sensor coil, comparing the thresholds of the *r*-*L* discharge waveform via a comparator to determine the inductance value, and evaluating if a target is approaching. This method is simple and popular. Due to the influence of temperature drift, however, a quantitative output is difficult to realize. Fericean [6] and Nabavi [15] proposed thermistor and differential coils to reduce temperature drift, respectively. These methods improve the IPS temperature characteristic but cannot produce a quantitative output because of the precision and consistency restriction in analog compensation or offsetting.

Digital measurement procedures include applying a sine wave excitation to a sensor coil, sampling voltage and current waveforms, using a Fourier transform algorithm to identify the phase difference between the voltage and the current, and calculating the inductance and resistance components of coil [7]. The inductance component is directly related to proximity distance; therefore, temperature drift can be reduced and quantitative output produced [16]. Digital signal processing is complicated; this process uses random-access memory (RAM) and process-control-based components such as micro-controller unit (MCU) or digital signal processor (DSP). The procedure may induce large volume and high power consumption; thus, its application in the aviation field is constrained [17].

Leons *et al.* [18] proposed a measurement method that applied pulse excitation to the sensor coil and obtained discharge waveform samples, and measured an integral average value of a discharge waveform for reducing the noise impact and calculated the distance using a one-dimensional look-up table. That system can measure distances in the range of 0∼5 mm at 89% accuracy under room temperature. However, the reference also indicated that “We also plan to study the accuracy of our design with modifications in temperature”.

This paper combines the characteristics of the analog and digital measurements; pulse excitation and sampling are commonly used for these techniques, respectively. Given the characteristics of the two methods, this study proposes a new method and processing circuit that obtains the discharge waveform samples of an IPS coil twice as well as separates the inductance and resistance components through discharge model calculation. The measurement errors caused by temperature drift are reduced, and a quantitative output is realized. This work also proposes a two-dimensional look-up table-based method, and online data calculation is simplified. Without the need for complex floating point computation, process control units are omitted, such as the MCU-class. The look-up table is effectively compressed by maximizing the distribution characteristics of the sample value. Furthermore, memory can be significantly decreased. A real-time built-in self-test (BIST) is designed with reference to the partition and analysis of abnormal sample data. This method has the advantages of the traditional analog and digital measurement methods; it also improves temperature stability, reliability, and real-time measurement. The servo system applies proportional-integral-differential (PID) control through the IPS quantitative output; this accelerates and stabilizes the process in addition to expanding the IPS application function.

## 2. System

### 2.1. Front-End Interface

**Figure 1:**
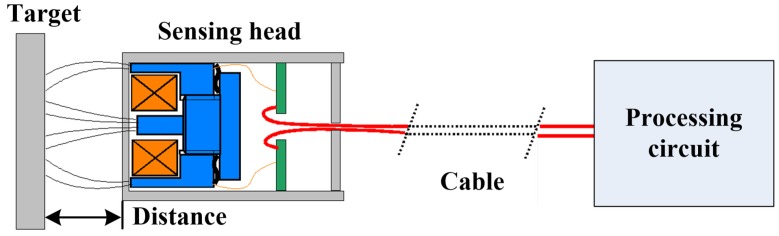
The schematic diagram of inductive proximity sensors.

IPS mainly consists of a sensing head and a processing circuit, as shown in Figure 1. The processing circuit can be either embedded into the sensing head or spread outside the sensing head via a cable connection. The distance between the sensing head and the target is calculated and outputted by driving and detecting the coil.

**Figure 2:**
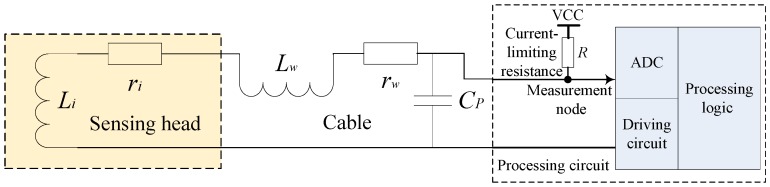
The equivalent circuit of the IPS.

In an equivalent circuit, as shown in Figure 2, $L_{i}$ is the inductance component of the coil in the sensing head, $r_{i}$ is the resistance component of the coil, $L_{w}$ is the equivalent distributed inductance of cable, $r_{w}$ is the equivalent distributed resistance of the cable, and $C_{P}$ is the equivalent parasitical capacitance. The general series resistance $r_{DC}$ (equal to $r_{i}$ + $r_{w}$) is 13.2 Ω based on the measurement of the sensing head in this paper (on the condition that 1.0 V at 1 kHz, The equipment of E4980A (Agilent, Santa Clara, CA, United States) is used, and the cable is 200.0 mm long). When the distance between the sensing head and the target is greater than 10.0 mm, the general series inductance $L_{S}$ (equal to $L_{i}$ + $L_{w}$) is 4.9 mH; when the distance is less than 0.1 mm, $L_{S}$ is approximately 9.4 mH. When an impedance analyzer (HP4195A, Agilent, Santa Clara, CA, United States) is employed to scan the sensing head, $C_{P}$ is 262.7 pF. The effect of distributed capacitance on the system parameter is considerably less than 1‰; the distribution parameter can be thus ignored.

Experimental data shows that $r_{DC}$ changes significantly with temperature but is weakly correlated with the distance between the sensing head and the target. Moreover, $L_{S}$ varies significantly with distance but is slightly correlated with temperature [1,7,8]; thus, $L_{S}$ reflects the distance between the sensing head and the target. The measurement is affected by cable length and temperature; as for the linear time-invariant parameter system, the influence of cable length on measurement is fixed and can be eliminated through calibration. The influence of temperature on measurement is dynamic; therefore, separating the resistance component $r_{DC}$ and the inductance component $L_{S}$ of the IPS coil is key to eliminating the effect of temperature variation on measurement precision.

**Figure 3:**
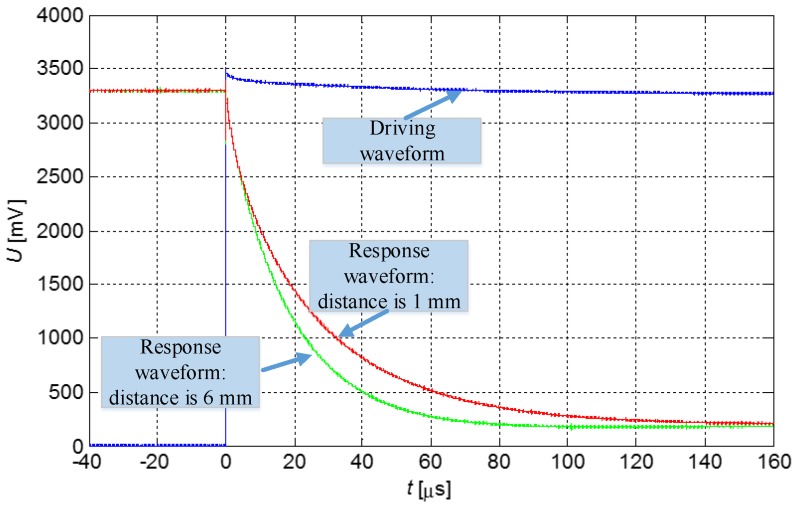
Driving and response waveforms of the sensing head.

The sensing head is connected to the processing circuit via a cable. When driving circuit generates an excitation of rising edge, a signal passes through current-limiting resistance and drives the sensing head to discharge. As shown in Figure 3, the discharge waveforms vary significantly in the time domain as the distance changes. Calculating and processing logic are employed to obtain a sample and to analyze the discharge waveform through an analog-to-digital converter (ADC). Subsequently, the distance information can be deduced.

**Figure 4:**
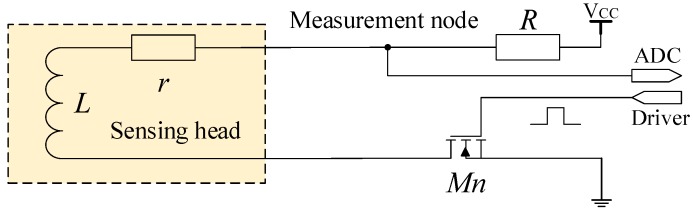
Driving and sampling of the integrative IPS.

This study focuses on a common integrative IPS; its processing circuit is embedded in the sensing head and is directly connected to the coil. The distribution parameter of the cable that connects the sensing head and the processing circuit can be neglected. The driving and sampling circuits are designed as shown in Figure 4.

Once the switch transistor $M_{n}$ is activated, the inductance *L* discharges slowly through *r* and *R* as shown in Figure 4. The voltage of the measurement node is determined as follows:

(1) $$U=\frac{V_{CC}}{R+r}\left[r+Re^{-\frac{R+r}{L}t}\right]$$

This paper presents a unique measurement method, the ADC in the processing circuit samples twice at $t_{1}$ and $t_{2}$. The samples $U_{1}$ at $t_{1}$ and $U_{2}$ at $t_{2}$ can be represented as follows:

(2) $$\begin{array}{c} \left\{\begin{array}{ccc} U_{1} & = & \frac{V_{CC}}{R+r}\left[r+Re^{-\frac{R+r}{L}t_{1}}\right]\\ U_{2} & = & \frac{V_{CC}}{R+r}\left[r+Re^{-\frac{R+r}{L}t_{2}}\right] \end{array}\right. \end{array}$$

Through equipment measurement mentioned above, the scopes of coil parameters are determined. When the target moves, the variation range of *L* is from 4.0 mH to 10.0 mH; when the temperature changes, the variation range of *r* is from 8 Ω to 20 Ω. The aforementioned scopes are regarded as the definition domain of (r,L).

When the sample value of the 12-bit ADC is applied to express $U_{1}$ and $U_{2}$, $V_{CC}$ is 4095 times the least significant bit (LSB) of ADC.

The discharge curve of the IPS is determined when the parameters take the mean value of the scope of resistance *r* and inductance *L*. $t_{1}$ is the moment in which the measurement node discharges to 60% $V_{CC}$, whereas $t_{2}$ is the moment in which the measurement node discharges to 30% $V_{CC}$. $t_{1}$ and $t_{2}$ are regarded as constants; at the two specific moments, ADC is controlled to conduct sampling twice. Based on the current-limiting condition, current-limiting resistance *R* takes a value of 300 Ω.

$V_{CC}$, $t_{1}$, $t_{2}$, and *R* are determined; *L* and *r* can be calculated by substituting $U_{1}$ and $U_{2}$ into Equation (2). The distance between the sensing head and the target can be indicated by *L*.

### 2.2. Construction of a Look-up Table

Sensor proximity status is obtained by building a look-up table and deriving the inductance value *L* from the sample $\left(U_{1},U_{2}\right)$. This process avoids complicated calculation; nevertheless, the following requirement should be fulfilled. Inductance value *L* can be determined through reasonable sampling $\left(U_{1},U_{2}\right)$, and the searching result is unique and correct. Thus, the following lemma should be met.

**Lemma 1.**  Equation (2) has one and only one solution.

**Proof.**  U(r,L) is a monotone increasing function about *r* and *L*. As U(r,L) is a monotone and continuous function, an inverse function exists, and Equation (2) generates a solution.

**Figure 5:**
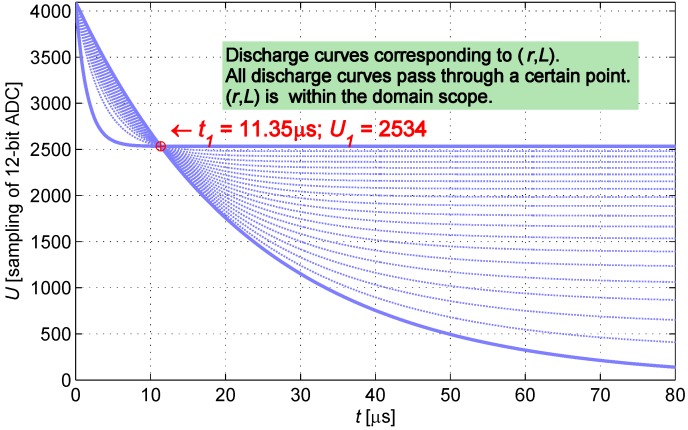
Cluster of curves passing through $\left(U_{1},t_{1}\right)$.

**Figure 6:**
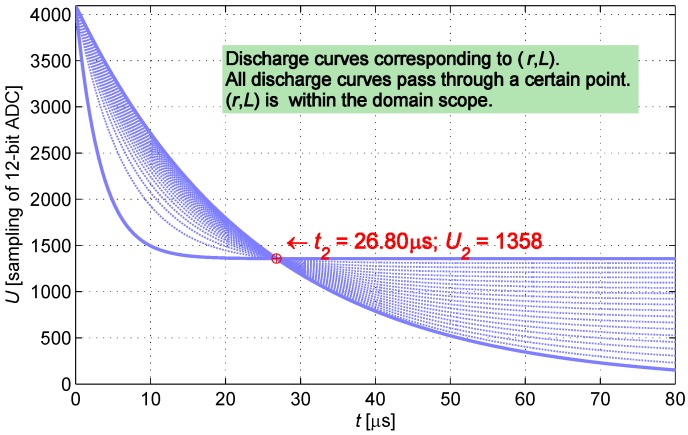
Cluster of curves passing through $\left(U_{2},t_{2}\right)$.

Given the value $\left(U_{1},t_{1}\right)$, *r* and *L* are subject to restriction conditions. Space ***p*** is defined as a set of $\left(r_{p},L_{p}\right)$, which enables function U(t) to pass through the point $\left(U_{1},t_{1}\right)$ as shown in Figure 5; space ***q*** is defined as a set of $(r_{q},L_{q}$), which enables function U(t) to pass through the point $\left(U_{2},t_{2}\right)$ as shown in Figure 6.

Hence, Equation (2) generates one and only one solution. ☐

**Figure 7:**
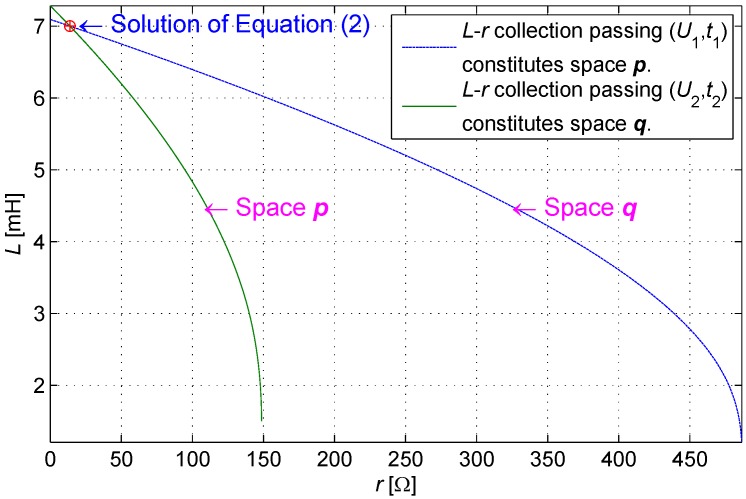
The intersection of spaces ***p*** and ***q*** as the solution of Equation (2).

The spaces ***p*** and ***q*** are drawn in *r*-*L* coordinates, as shown in Figure 7. The intersection of the two curves is the solution of the equation because it passes through $\left(U_{1},t_{1}\right)$ and $\left(U_{2},t_{2}\right)$ synchronously. L(r) is monotonic; therefore, a maximum of only one intersection exists. Equation (2) thus produces only a single solution at most.

Equation (2) is a transcendental equation, and obtaining an analytical solution is difficult. According to Lemma 1, a numerical solution that satisfies an engineering accuracy requirement can be obtained through iteration.

Subjecting to (s.t.) the given conditions, the values of *L* and *r* that minimize (min) the below formula are the numerical solution of Equation (2):

(3) $$\begin{array}{c} \begin{array}{ccc} \min & \; & {\left[U_{1}-U\left(L,r,t_{1}\right)\right]}^{2}+{\left[U_{2}-U\left(L,r,t_{2}\right)\right]}^{2}\\ \text{s.t.} & \; & r>0\;,\;L>0\\ & \; & 0<t_{1}<t_{2}\;,\;t_{1}\;\;\mathrm{and}\;\;t_{2}\;\;\mathrm{are}\;\mathrm{determined} \end{array} \end{array}$$

The digital solution of (r,L) that corresponds to $\left(U_{1},U_{2}\right)$ is obtained by traversing the sample value $\left(U_{1},U_{2}\right)$, and a look-up table is established. In practice, the value of *L* is obtained with $U_{1}$ and $U_{2}$ to determine the target proximity status. The optimization algorithm can ensure that the precision of the solved *L* meets the requirement. *L* reflects the distance between the sensing head and the target.

### 2.3. Compression of the Look-up Table

For instance, the size of a complete two-dimensional look-up table is $2^{12}\times 2^{12}$ units when the 12-bit ADC is used; this size indicates poor practicability. Therefore, an effective method to compress the look-up table should be developed to ensure the practicability of this method. The look-up table can be compressed effectively by analyzing the distribution characteristics of the sample value $\left(U_{1},U_{2}\right)$ in a real circuit.

In practice, $\left(r_{i},L_{i}\right)$ is calculated by the given $\left(U_{1i},U_{2i}\right)$ value. The solution scope can be narrowed by the restriction of the physical model.

Supposing $U_{1}<U_{2}$ (when $t_{1}<t_{2}$), the monotony of U(t) changes. Thus, the equation solution is negative in value and is physically unreasonable.

Equation (1) is converted into an inverse function of *U* for *L*:

(4) $$L=-\frac{(R+r)t}{\mathrm{lg}\left[\frac{U}{4095R}\left(R+r\right)-\frac{r}{R}\right]}$$

*L* belongs to a positive real domain, so the following constraint should be fulfilled:

(5) $$0<\left[\frac{U}{4095R}\left(R+r\right)-\frac{r}{R}\right]<1$$

That means the sample value should meet the following constraint:

(6) $$\frac{4095r}{R+r}<U<4095$$

If $\left(U_{1i},U_{2i}\right)$ is improperly given, the solution $\left(r_{i},L_{i}\right)$ may consist of a negative or even a complex number. In fact, the sampled $\left(U_{1},U_{2}\right)$ is certainly reasonable. A restriction condition exists between $U_{1}$ and $U_{2}$ such that (r,L) belongs to a positive real domain. The intersection of two curves depicted in Figure 7 is observed at the first quadrant of the real domain coordinate. Beyond the restriction condition, $\left(U_{1},U_{2}\right)$ will not be obtained through practical sampling and need not be recorded in the look-up table.

Given samples $U_{1}$ at $t_{1}$ and $U_{2}$ at $t_{2}$, the following equation is obtained.

(7) $$\begin{array}{c} \left\{\begin{array}{ccc} L & = & -\frac{\left(R+r\right)t_{1}}{\mathrm{lg}\left[\frac{U_{1}}{4095R}\left(R+r\right)-\frac{r}{R}\right]}\\ L & = & -\frac{\left(R+r\right)t_{2}}{\mathrm{lg}\left[\frac{U_{2}}{4095R}\left(R+r\right)-\frac{r}{R}\right]} \end{array}\right. \end{array}$$

Furthermore, the constraint relationship of $U_{1}$ and $U_{2}$ is obtained.

(8) $${\left[\frac{U_{1}}{4095R}\left(R+r\right)-\frac{r}{R}\right]}^{t_{2}/t_{1}}=\left[\frac{U_{2}}{4095R}\left(R+r\right)-\frac{r}{R}\right]$$

If a set of $\left(U_{1},U_{2}\right)$ satisfies both Equations (6) and (8), then Equation (2) generates a solution with a positive real number.

**Figure 8:**
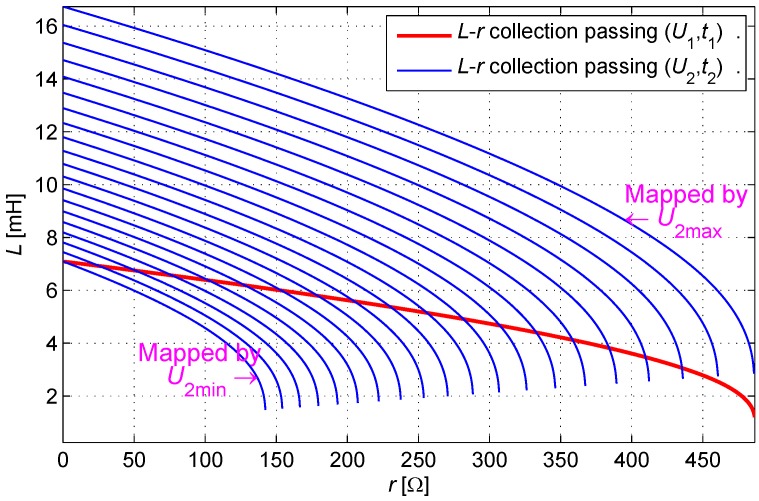
Cluster of curves mapped by $U_{2}$, which have positive real intersections with a given curve mapped by $U_{1}$.

A restriction condition of $U_{2}\left(r\right)$ is obtained when $U_{1}$ is given. $U_{2}\left(r\right)$ is proven as a monotone function, and the range of its values is determined by the definition domain of *r*. Under the range of $U_{2}\left(r\right)$ in combination with the given $U_{1}$, Equation (2) generates positive real number solutions, as shown in Figure 8.

The essence of a look-up table lies in the mapping of the points from the $U_{1}-U_{2}$ coordinate to the r-L coordinate. All points in the r-L coordinate can be mapped into the $U_{1}-U_{2}$ coordinate. Some points in the $U_{1}-U_{2}$ coordinate can be mapped back into the r-L coordinate. A few of the other points in the $U_{1}-U_{2}$ coordinate will fall under the other quadrants (negative number) or a four-dimensional complex space (complex number) when mapping to the r-L coordinate.

Through equipment measurement mentioned in Subsection 2.1, the scopes of coil parameters are determined. The engineering parameter definitions of the coil are r∈[8.0,20.0]Ω and L∈[4.0,10.0] mH. The definition domain of $U_{1}$ can be obtained based on the restriction condition of Equation (1) and the monotony of U(r,L), as expressed in Equation (9):

(9) $$\begin{array}{c} \left\{\begin{array}{ccc} U_{1\min} & = & \frac{4095}{R+r_{\min}}\left[r_{\min}+Re^{-\frac{R+r_{\min}}{L_{\min}}t_{1}}\right]\\ U_{1\max} & = & \frac{4095}{R+r_{\max}}\left[r_{\max}+Re^{-\frac{R+r_{\max}}{L_{\max}}t_{1}}\right] \end{array}\right. \end{array}$$

Given the restriction condition of Equation (8), a range of $U_{2}$ values corresponding to *r* within a physical changing range can be determined with each given $U_{1}$, as expressed in Equation (10):

(10) $$\begin{array}{c} \left\{\begin{array}{ccc} U_{2\min} & = & 4095R\frac{{\left[\frac{U_{1}}{4095R}\left(R+r_{\min}\right)-\frac{r_{\min}}{R}\right]}^{t_{2}/t_{1}}+\frac{r_{\min}}{R}}{\left(R+r_{\min}\right)}\\ U_{2\max} & = & 4095R\frac{{\left[\frac{U_{1}}{4095R}\left(R+r_{\max}\right)-\frac{r_{\max}}{R}\right]}^{t_{2}/t_{1}}+\frac{r_{\max}}{R}}{\left(R+r_{\max}\right)} \end{array}\right. \end{array}$$

**Figure 9:**
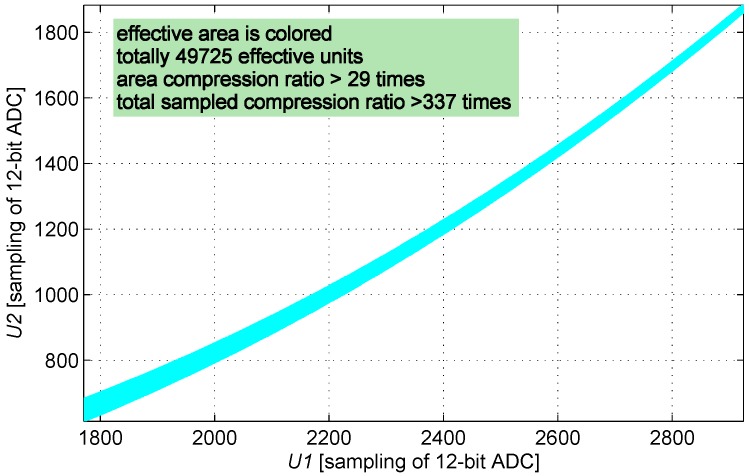
Effective table area according to the domain of the engineering definition.

The definition domain of $U_{2}$ is determined in sequence by traversing the definition domain of $U_{1}$, and the definition domain of $\left(U_{1},U_{2}\right)$ is determined as shown in Figure 9.

The size of the look-up table is compressed from 4096 × 4096 into 49,725 units; therefore, the look-up table is compressed 337 times.

### 2.4. Real-Time BIST

In general, the traditional scheme implements the BIST process after the system powers on and enters the working state. Therefore, BIST detection cannot be performed simultaneously during the system working process.

The position information is obtained by analyzing and calculating the sample value of the discharge waveform. When coil disorder or driving circuit failure occur and the sample value is beyond the definition domain, failure statuses and modes can be assessed and outputted in real time.

**Figure 10:**
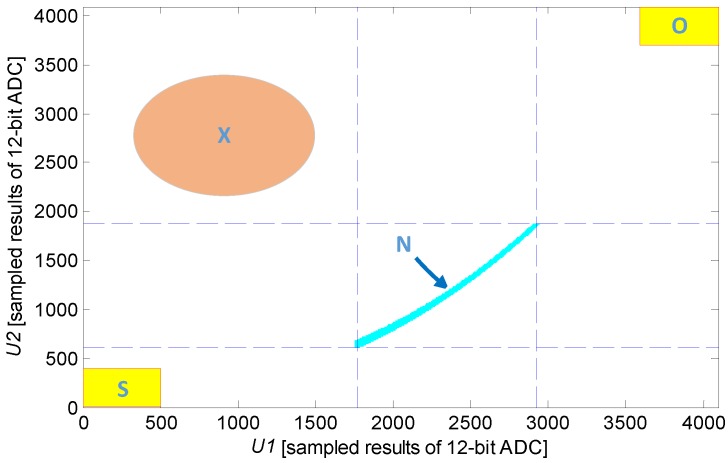
Built-in self-test vector diagram.

As mentioned previously, look-up table information is established in the parameter scope of the IPS coil. Sample datum that are not recorded in this table can be obtained during IPS system failure. Different failure modes can be obtained without the need for an additional circuit by analyzing the unrecorded sample data. As shown in Figure 10, if the sample value falls under domain **N**, then the system searches for position information in the look-up table. If the sample value falls under domain **S**, then the system evaluates the coil as short-circuited; if the sample value falls under domain **O**, then the system regards the coil as an open circuit or as subject to driving circuit failure.

The increased accumulation of engineering applications in the future will be conducive to improving the integrality of failure modes and to establishing the relationship of these failure modes with the **X** region. In this case, the area covered by BIST expands, the lifetime of IPS can be forecasted, and an effective online intelligent monitoring function can be established for the sensor IPS.

## 3. Implementation

**Figure 11:**
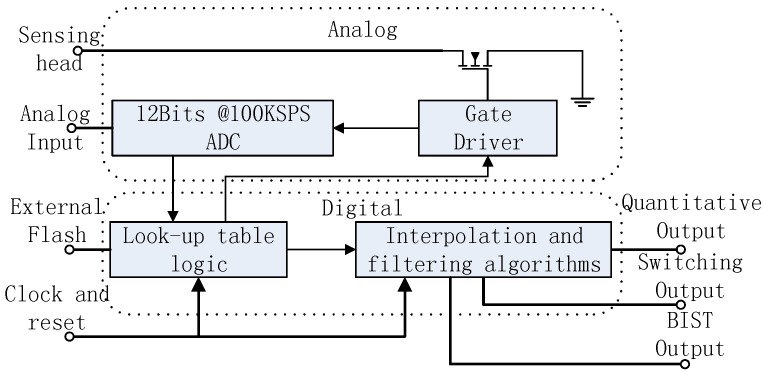
Block diagram of the integrative micro-assembly module.

As shown in Figure 11, the integrative micro-assembly module of the processing circuit includes a gate driver, look-up table logic, and the interpolation and filtering algorithms. On the excitation of the gate driver, the sensing head generates an analog input for ADC. Given the sample value, the look-up table logic calculates a proximity distance. The interpolation algorithm extracts an additional linear resolution of the proximity distance. The filtering algorithm is used to analyze abnormal samples and to generate outputs in the following format: the quantitative output is exhibited by a universal asynchronous receiver/transmitter (UART); the switching output is exhibited by a one-bit digital signal, and the BIST output is exhibited by a two-bit digital signal. The module requires external conditions, including a current-limiting resistor, probe assembly (coil), external analog-filtering circuit, flash, oscillator, reset, and power circuit.

**Figure 12:**
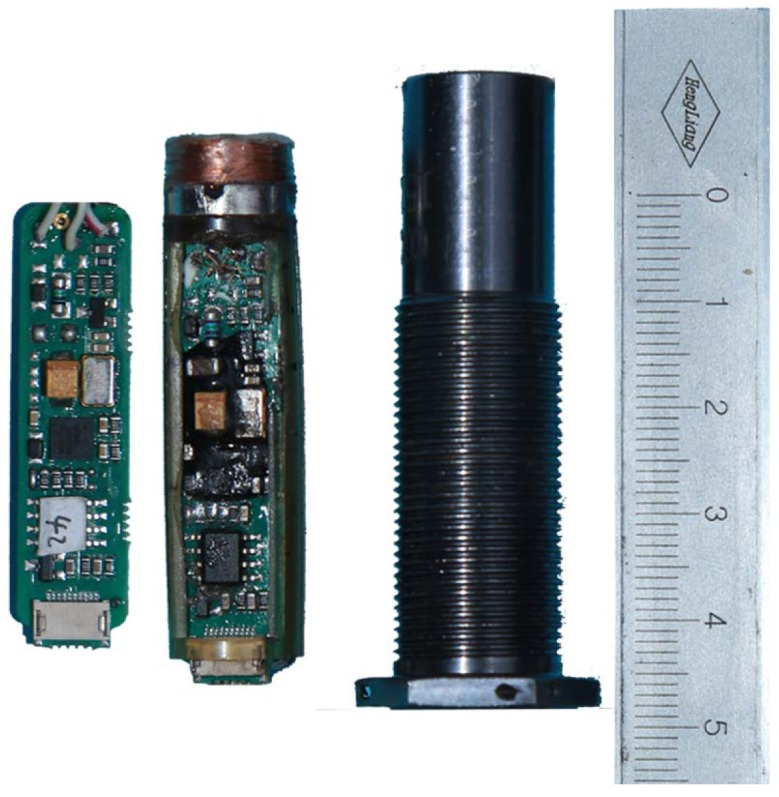
The engineering prototype of the inductive proximity sensor.

The engineering prototype of the IPS based on integrative micro-assembly is depicted in Figure 12. The IPS coil is a ϕ5 mm × 12 mm cylinder that is wound by ϕ0.14 mm of enameled wire. The core is integrated into a processing circuit that is composed of two circuit boards: the power board is inputted with an external power supply and outputs the voltages for the digital board; the digital board calculates and outputs information on distance and BIST. Those two circuit boards are integrated into a threaded barrel that measures ϕ14 mm × 59 mm.

This study is implementing a procedure for the realization of an application-specific integrated circuit (ASIC) used to substitute for the micro-assembly module. Besides, system integration contributes to the improvement of stability.

## 4. Results And Discussion

This section establishes the relationship between the sample value $\left(U_{1},U_{2}\right)$ and the inductance component *L* of the IPS coil based on the discharge model. Moreover, a quantitative error analysis is conducted on the look-up table. A prototype experimental platform is designed and built as described in this section. The relationship between the inductance component *L* of the IPS coil and proximity distance is established with this platform, and the look-up table of proximity distance that is related to sample value $\left(U_{1},U_{2}\right)$ is constructed. Finally, the look-up table is programmed into the IPS engineering prototypes, and a testing procedure is implemented.

**Figure 13:**
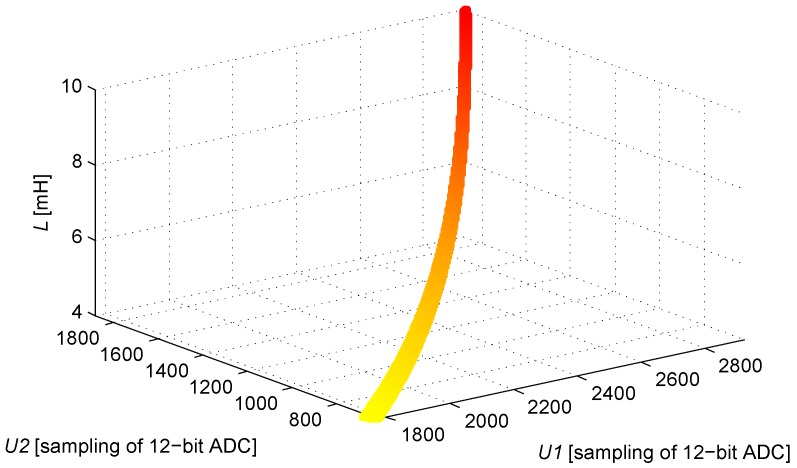
The solution of function $L(U_{1},U_{2})$.

According to the definition of the $\left(U_{1},U_{2}\right)$ domain illustrated in Figure 9, the corresponding *L* is solved by applying Equation (3), and the look-up table is established. An image of the function $L(U_{1},U_{2})$ within the definition domain is obtained by taking *L* as the Z-axis, as shown in Figure 13.

The quantitative error evaluation testifies that the maximum quantization error of *L* is 12.08 μH. This error is detected when $U_{1}=2925$, $U_{2}=1882$, and L=9984.51mH. The IPS system reaches a theoretical resolution of 1.21‰ when the system uses a 12-bit ADC.

This study emphasizes the $L_{S}-r_{DC}$ parameters. It also establishes the relationship of $L_{S}-r_{DC}$ with proximity distance through an experimental method given that production and assembly are inconsistent and that the difference of coil is less than 2%, which is far beyond the theoretical resolution [19,20].

**Figure 14:**
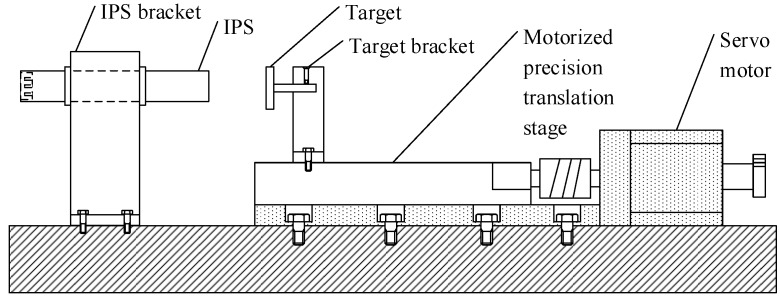
Experimental platform.

An experimental platform is designed and built to control the distance between the sensing head and the target automatically, as shown in Figure 14. According to an optics motorized precision translation stage, the re-orientation accuracy of the experimental platform is ±20 μm, which is ensured by the related metrology accreditation.

**Figure 15:**
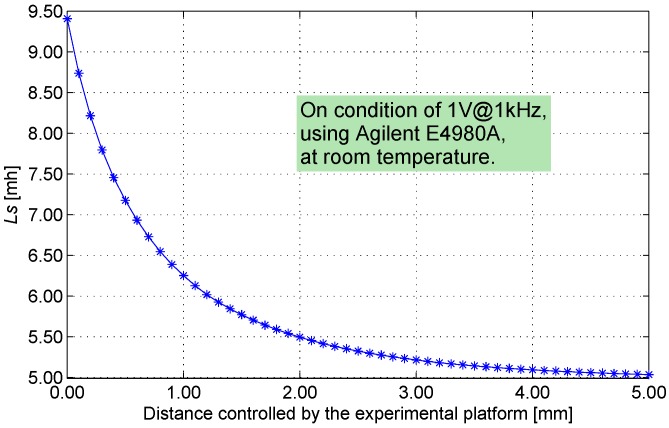
Distance *vs.* $L_{S}$.

The materials of the target are related to the magnetic environment of the IPS working process. The IPS can detect a target of steels. Moreover, slight sensitivity differences are observed among various alloy steels. The alloy steel of 0Cr17Ni5Cu4Nb is commonly selected as the material of the target in the industry; thus, this material comprises the target on the experimental platform in this study. The target is produced in the shape of a coin that is 16.0 mm in diameter and is 2.5 mm thick. The IPS assembled with only a coil component is installed on the platform; this IPS is used to establish the relationship of $L_{S}-r_{DC}$ with proximity distance. Under this circumstance, the relationship of the observed distance (between the sensing head and the target) and the $L_{S}$ of the coil is determined, as shown in Figure 15.

The constraint relationship of distance and $L_{S}$ is nonlinear [7]; therefore, the measurement precision in relation to distance is uneven. The effective resolution of IPS worsens if distance increases [18]. Sensors of this type are constrained by this characteristic, and this feature is applicable only in short-distance (less than 8 mm) detection.

According to the calibrated data, the distance between the sensing head and the target can be searched from the sample values $\left(U_{1},U_{2}\right)$ when the inductance value of the look-up table is replaced with distance.

To conduct the temperature experiment, the servo motor on the mechanical part of the experimental platform is connected to the control box via cables. In this experiment, the mechanical part is placed into a high-temperature box. We can set the distance between the IPS and the target through a human-machine interface of the control box outside of the high-temperature box.

**Figure 16:**
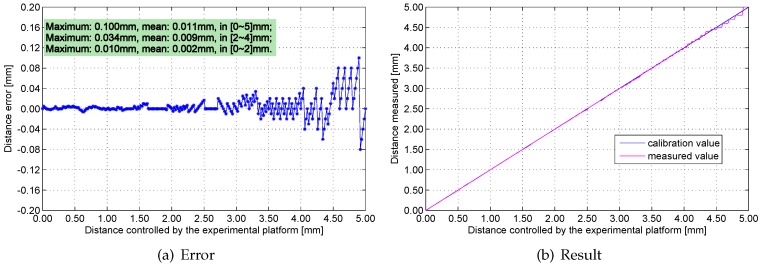
Measurement errors and results at 20 °C.

**Figure 17:**
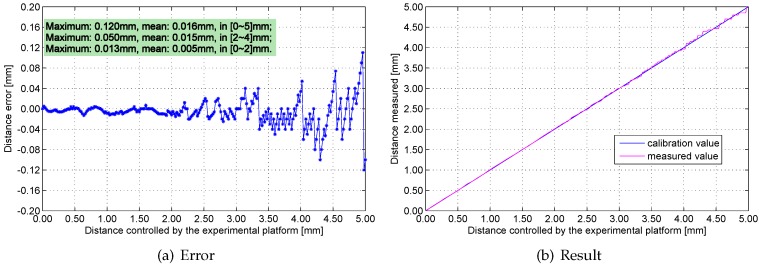
Measurement errors and results at 50 °C.

**Figure 18:**
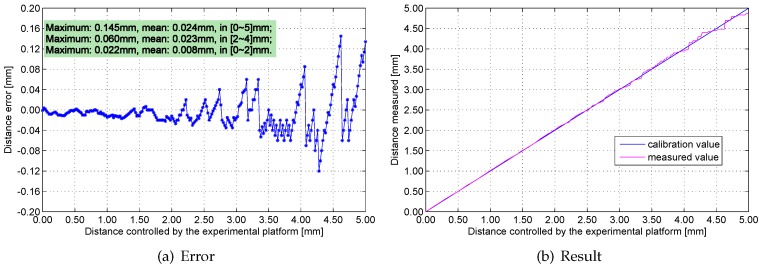
Measurement errors and results at 80 °C.

**Figure 19:**
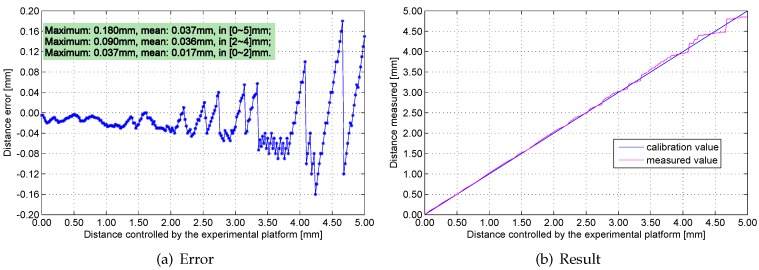
Measurement errors and results at 110 °C.

Figure 16, Figure 17, Figure 18 and Figure 19 show the measurement errors and results of the engineering prototype at 20 °C, 50 °C, 80 °C and 110 °C respectively. The experimental data indicates two rules: first, the maximum and mean errors increase as temperature increases. They are mainly caused by the temperature drift of the experimental platform and the inductance component. Second, the error increases with distance because the sensitivity of the sensing head drops rapidly if distance increases. The maximum error is 0.18 mm, which is below 4%.

**Figure 20:**
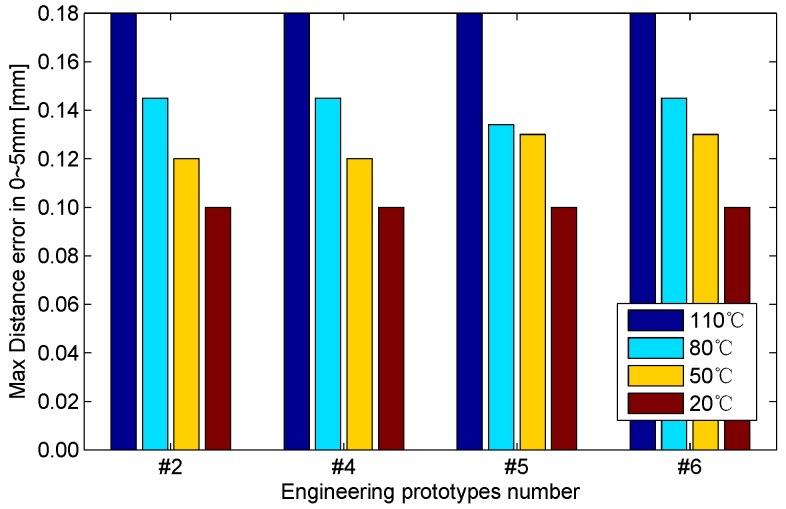
Max distance error of the four engineering prototypes at 0–5 mm.

Four engineering prototypes are included in the aforementioned experiment. No significant difference is observed among these prototypes based on the analysis of the experimental data. The max distance error of the four engineering prototypes is detected at 0–5 mm, as shown in Figure 20.

**Figure 21:**
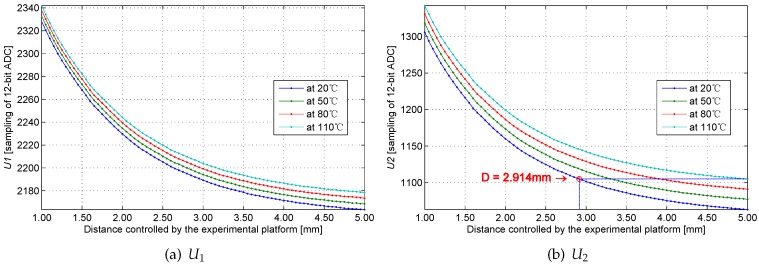
ADC samples $U_{1}$ and $U_{2}$ at different temperatures.

Figure 21a,b exhibits the ADC samples $U_{1}$ and $U_{2}$ at different temperatures. The smoothness and monotony of the curves suggest that the system sampling is stable and that background noise is controlled below 1 LSB of ADC.

If the temperature effects on the sensing head are neglected, the four waveforms in Figure 21 would be coincident, and the twice-sampling method can be simplified into a single sampling method. As shown in Figure 21b, if the distance reaches 5 mm at 110 °C, the curve generated at 20 °C could explain the curve established at 110 °C (if the temperature effects are neglected). The computing distance value is 2.914 mm, and the error is 2.086 mm.

Unlike the study conducted by Leons *et al.* [18], this study used different processing circuits that can effectively control background noise in addition to obtaining samples of the discharge waveform of IPS coils twice; the inductance and resistance components are separated through the calculation process based on a two-dimensional look-up table, and the temperature drift is reduced. Experimental data shows that, if the temperature drift of the coil is disregarded, the temperature variance in the applicable scope strongly affects system precision.

Therefore, obtaining the discharge waveform samples of the IPS coil twice as well as separating the inductance and resistance components are the key points in reducing temperature drift and improving the precision of the IPS system.

The system measurement is implemented during the activation of the controlled switch; the external jamming out of the measurement window is thus unrelated to the measurement result. The system, therefore, has a unique anti-jamming capability.

**Figure 22:**
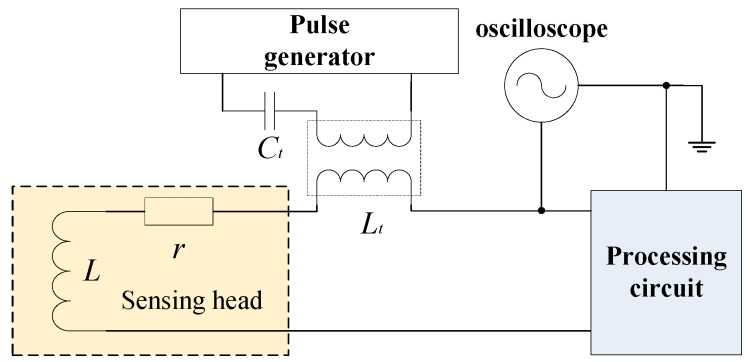
Jamming input method.

This study established a testing framework that is similar to the voltage spike test of category A in section 17 of DO-160F. The engineering prototype has passed the related test of DO-160F, and the result confirms that input jamming is inadequate to cause a temporary failure. Instead of the common mode jamming applied in the test of DO-160F, differential mode jamming is input into the IPS discharge circuit. As shown in Figure 22, a pulse generator creates 10 V of a 200 Hz square wave and inputs jamming into the system via capacitance $C_{t}$ and transformer $L_{t}$. $C_{t}$ is 1 μF. The inductance in series with discharge circuit is 5 μH.

**Figure 23:**
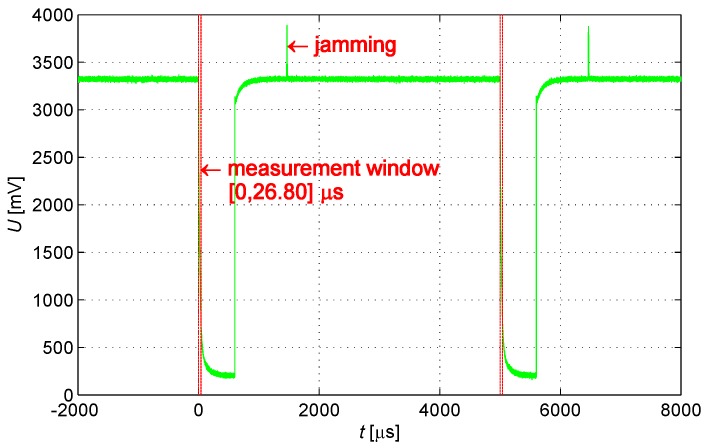
An anti-jamming process.

In the test, an oscilloscope is used to observe discharge waveforms with jamming, as shown in Figure 23; the jamming input is enough to cause a temporary failure. Even though jamming beyond the measurement window alters the coil recharge status and then the status is recovered quickly through discharge, the jamming effect does not influence the working status of the subsequent measurement window. Therefore, a narrow measurement window improves the anti-jamming capability.

Some public released references were selected to make a comparison, as they have the similarities with this study in the following aspects: they have the same application background in the field of aviation; they are all integrative IPSs and have the similar parameters; they all avoid using process-control-based components. Table 1 presents the comparison and highlights the benefits of this study.

**Table 1:** Comparison of this work with related studies.

	This work	[17]	Honeywell ZS-00305 [21]
Temperature drift reduction	Self-adaptive, compensation is unnecessary	Customized thermal resistance, compensation is required	Unknown
Measurement method	Analog-digital mixed	Analog	Analog
Guaranteed actuation distance	4.50 ± 0.20 mm	3.28 ± 0.20 mm	4.00 ± 0.50 mm
Quantitative output	Yes	No	No
Built-in self-test	Yes, real-time	Yes, non-real-time	Yes, non-real-time
Electromagnetic compatibility	Narrow measurement window, strong anti-jamming capability	No measurement window, weak anti-jamming capability	No measurement window, weak anti-jamming capability
Input current (at 28 V)	6 mA	4 mA	10 mA
MCU or DSP	Non-adoptive	Non-adoptive	Non-adoptive

This work obtains two samples of the pulse discharge waveform of the IPS coil and separates the two variables $L_{S}$ and $r_{DC}$ through modeling calculation. Precision is improved, and the quantitative output of proximity distance is realized. The two-dimensional look-up table method reduces online computational complexity. Thus, process-control-based components, such as MCU, are saved.

Through an analysis of the characteristics of the look-up table and the exploitation of the physical parameter constraint of the coil, the look-up table is compressed 337 times, the processing circuit is simplified, and power consumption is reduced. This study establishes an abnormal sample partition for data beyond the look-up table, extracts IPS failure modes, and establishes a real-time BIST mechanism. Two failure models are assessed, namely, the short-circuit and open-circuit models. Based on more engineering application accumulation in future, BIST covering area will be comprehensive, and healthy online intelligent monitoring function will be realized [21].

In this work, the sample waveform includes the static component of the coil, which does not change with temperature or proximity status [22]. Under its influence, the dynamic range of ADC cannot be maximized [23]. The two sets of coils with symmetric parameters embedded into the sensor are proposed for future studies. The coil in front is close to the target, and can extract proximity variation; the rear coil is far from the target, and can be regarded as a parameter reference. The DC resistance of the rear coil changes with temperature as with the coil in front. System sensitivity and resolution can be improved by detecting the discharge waveform extracted from the differential circuit [24].

## 5. Conclusions

The IPS structure and test method were researched in this study. To reduce temperature drift and to improve the test performance, a new mixed method of analog and digital testing was presented that conducts twice sampling of the discharge waveform. Through discharge modeling calculation, the inductance and resistance components are separated, the measurement error caused by temperature drift is reduced, and the quantitative output is realized, moreover, the IPS application scope is expanded. The establishment and application of the two-dimensional look-up table reduce system online computational complexity. It also saves process-control-based components and improves measurement stability, reliability, and real-time performance. By analyzing the exploitation of the physical parameter constraints of the sensing head, the size of the look-up table is compressed 1/337 of the original processing circuit. Furthermore, power consumption is reduced. The realization of the real-time BIST extends the IPS detection capabilities.

Experiment data and comparison of related products indicate that the aforementioned method is especially suitable for fulfilling the high-performance requirements, such as displacement measurement in the fields of aerospace and aviation.
